# Low density parasitaemia, red blood cell polymorphisms and *Plasmodium falciparum *specific immune responses in a low endemic area in northern Tanzania

**DOI:** 10.1186/1471-2334-9-69

**Published:** 2009-05-21

**Authors:** Seif Shekalaghe, Michael Alifrangis, Charles Mwanziva, Anders Enevold, Steve Mwakalinga, Humphrey Mkali, Reginald Kavishe, Alphaxard Manjurano, Robert Sauerwein, Chris Drakeley, Teun Bousema

**Affiliations:** 1 Department of Medical Microbiology, Radboud University Nijmegen Medical Centre, Nijmegen, The Netherlands; 2 Kilimanjaro Christian Medical College, Moshi, Tanzania; 3 Centre for Medical Parasitology at the Department of International Health, Immunology and Microbiology, University of Copenhagen, and at the Department of infectious Diseases, Copenhagen University Hospital (Rigshospitalet), Copenhagen, Denmark; 4 Joint Malaria Programme, Moshi, Tanzania; Kilimanjaro Christian Medical Centre, Moshi, Tanzania; Department of Infectious and Tropical Diseases, London School of Hygiene and Tropical Medicine, London, UK

## Abstract

**Background:**

Low density *Plasmodium falciparum *infections, below the microscopic detection limit, may play an important role in maintaining malaria transmission in low endemic areas as well as contribute to the maintenance of acquired immunity. Little is known about factors influencing the occurrence of sub-microscopic parasitaemia or the relation with immune responses.

We investigated possible associations between the occurrence of sub-microscopic *P. falciparum *parasite carriage and antibody responses to the asexual stage antigens, G6PD deficiency and α^+^-thalassaemia in 464 subjects from a low endemic area in northern Tanzania.

**Methods:**

We used samples collected from two cross sectional surveys conducted during dry and wet season in 2005. Submicroscopic parasitaemia was detected by using quantitative nucleic acid sequence based amplification (QT-NASBA). Genotyping for G6PD and α^+^-thalassaemia were performed by high throughput PCR; the prevalence and level of total IgG antibodies against MSP-1, MSP-2 and AMA-1 were determined by ELISA.

**Results:**

Compared to parasite free individuals, individuals carrying sub-microscopic densities of *P. falciparum *parasites had significantly higher median antibody levels to MSP-1 (p = 0.042) and MSP-2 (p = 0.034) but not to AMA-1 (p = 0.14) while no clear relation between sub-microscopic parasite carriage and G6PD deficiency or α^+^-thalassaemia was observed.

**Conclusion:**

Our data suggest a role for sub-microscopic parasite densities in eliciting or maintaining humoral immune responses without evidence for a modulating effect of G6PD deficiency or α^+^-thalassaemia.

## Background

*Plasmodium falciparum *is responsible for the majority of malaria attributed deaths in sub-Saharan Africa although the parasites are also frequently present in the human circulation without causing malaria symptoms. Individuals in malaria-endemic areas can carry microscopically detectable levels of *P. falciparum *asymptomatically[1,2]. Moreover, recent molecular detection techniques have suggested the presence of a much greater proportion of asymptomatic infections below the microscopic threshold than previously believed [3,4]. Sub-microscopic infections have been primarily studied in areas of low and seasonal malaria transmission [3-6]. Here, it has been shown that sub-microscopic infections can persist for several months [3,5], produce gametocytes [5] and, despite low gametocyte concentrations in the infected individual, contribute to the transmission of malaria to mosquitoes [7-9]. Sub-microscopic infections may therefore play a role in maintaining malaria transmission in areas of low malaria endemicity. Despite their potential importance, little is known about factors influencing the occurrence of sub-microscopic parasitaemia and whether their presence may be associated with protective immune responses. However, long-term asymptomatic carriage of parasites at microscopic densities has been associated with protective immunity against subsequent clinical malaria attacks [10,11]. Despite indications from an experimental study showing that exposure to ultra low-dose infections may elicit protective immunity [12], there have been no field studies confirming the capacity of sub-microscopic infections to elicit or maintain immune responses.

Microscopically detected parasite carriage has been associated with several red blood cell polymorphisms, such as α^+^-thalassaemia, sickle cell trait and glucose 6 phosphate dehydrogenase (G6PD) deficiency [13,14]. In α^+^-thalassaemia, one gene of the two α-globin genes on each chromosome 16 is deleted and the deficiency has been associated with protection against severe [15,16] and mild malaria [16,17]. G6PD deficiency is a common chromosome x-linked red blood cell enzymopathy with several polymorphisms arisen from mutations in the G6PD gene. In Africa, a single point-mutation leads to the variant G6PD A with almost identical enzyme activity as the normal type (G6PD B), and a second point-mutation leads to the G6PD A- variant with highly reduced enzyme activity [18]. Similar to α^+^-thalassaemia, G6PD deficiency has been associated with protection against severe [19,20] and mild malaria [18,19,21]. Both α^+^-thalassaemia [22] and G6PD deficiency [23] may also protect against asymptomatic carriage of microscopically detectable levels of parasites although other studies did not find such associations [24,25]. The effect of red blood cell polymorphisms on sub-microscopic parasite carriage is unknown. Since these polymorphisms may not protect against initial infection but rather result in a slower parasite growth rate, as a consequence of a reduced parasite multiplication [26] or increased clearance of infected red blood cells [27], we hypothesize that the prevalence of sub-microscopic parasite carriage is higher in α^+^-thalassaemic and G6PD deficient individuals while that of high density parasitaemia is reduced.

Here, we investigate for possible associations between sub-microscopic *P. falciparum *parasite carriage, red blood cell polymorphisms and antibody responses to the asexual stage antigens that were recently explored as indicators of exposure to parasite antigen[28]: Merozoite Surface Protein (MSP)-1, MSP-2 and Apical Membrane Antigen (AMA)-1. The study was conducted in a population in northern Tanzania where the vast majority of parasite carriage occurs below the microscopic threshold for detection [4].

## Methods

### Study site and survey design

We utilized samples collected from a previously published study [4]. Briefly, two all age cross-sectional surveys were conducted during the dry and wet seasons (April and August, respectively) in 2005 in the villages Msitu wa Tembo, Kiruani and Magadini in the Lower Moshi area of northern Tanzania (latitude 3°33'-3°44's; longitude 37°17'-37°24'E). The area is characterised by low malaria transmission intensity with an entomologic inoculation rate of ~2.3 infectious bites per person per year (95% CI 0.7–9.9) [29]. A previous study estimated a malaria incidence in the study area of 38.4 episodes per 1000 person-years at risk [29]. Participants were selected using village census lists that were created for this study and computer randomized tables. Individuals were individually selected and invited to a central point in the village for sampling. No individuals provided more than one sample (i.e. both in the wet and dry season survey). In 2005, there was very low rainfall and we observed no marked seasonality or differences between the two cross-sectional surveys in parasite carriage [4]. Therefore we combined the data from both surveys. The study protocol was approved by the ethical committees of both the Tanzanian National Institute of Medical Research (NIMR/HQ/Vol.IX/343) and the London School of Hygiene & Tropical Medicine.

### Data collection, sample processing and analysis

A questionnaire was used to collect information on demographic, anthropometric and general health indicators. A single EDTA blood sample of approximately 200 μL was obtained from each participant by finger prick. This sample was used for i) preparation of blood smears for microscopy; ii) nucleic acid extraction for PCR and quantitative nucleic acid sequence based amplification (QT-NASBA); iii) plasma collection for immunological assays. Out of the total of 2752 individuals surveyed, 464 individuals were randomly selected using computer randomized tables from three age strata (below 5, 5–15 and above 15 years) in the ratio 1:1:1. No individuals were recruited more than once. From these individuals, nucleic acids were extracted from a 50 μL blood sample as described by Boom *et al. *[30]. The first part of the extraction was done in the field following the original guanidine isothiocyanate (GuSCN) RNA extraction method [30] until the nucleic acids were bound to silica dioxide particles. The remainder of the extraction was completed in the laboratory and RNA/DNA samples were stored at -20°C until analysis. QT-NASBA parasite detection was performed as described elsewhere using NucliSens Basic kits for amplification [31]. The forward primer was 5'-GTCATCTTTCGAGGTGACTT-3' (nucleotides 1136 to 1155); the reverse primer was 5'-AATTCTAATACGACTCACTATAGGGAGAAGGAACTTTCTCGCTTGCGCGAA-3' (T7 promoter sequence, linker, and nucleotides 1216 to 1235); the Pf18S molecular beacon was 5'-6-carboxyfluorescein-CGATCGGAGAAATCAAAGTCTTTGGG-CGATCG-dimethylaminoazosulfonic acid-3' (molecular beacon stem of 6 paired nucleotides and nucleotides 1182 to 1201). The time to positivity, i.e., the time point during amplification at which the number of target amplicons detected became higher than the mean for three negative controls plus 20 standard deviations, was calculated [32]. The detection limit of the QT-NASBA is 20 parasites/mL and parasite concentrations in test samples were determined using a standard ring stage parasite dilution series that was included in each run [32].

Genotyping for G6PD deficiency was performed by screening human DNA for single nucleotide polymorphisms in the G6PD gene (G202A) by a simple high throughput method using PCR followed by detection using sequence specific oligonucleotide probes (SSOPs) and ELISA based technology [33]. Individuals with no G202A mutation were classified G6PD B, heterozygotes for the G202A mutation were classified G6PD A and homozygote or hemizygote (males) for the G202A mutation were classified G6PD A-. The prevalence of α^+^-thalassaemia was determined by detection of the African α-globin deletion, α^3.7^, by PCR as described by Liu *et al. *[34].

### ELISA for the MSP-1, MSP-2 and AMA-1

IgG antibodies against blood stage malaria antigens were detected by indirect ELISA, as previously described [28] using recombinant MSP-1_19 _(Wellcome genotype); MSP-2 (3D7), AMA-1 (3D7), which were produced as described previously [35,36]. Briefly, flat bottom 96-well plates [Immulon 4HBX, Thermo] were coated overnight with 50 μL of 0.5 mg/mL dilution of the specific antigen. After washing with PBS-0.05% Tween [(PBS-T), 200 μL of blocking buffer (1% skimmed milk in PBS-T) was added for 3 hours at room temperature. After washing, plasma samples were added in duplicate at a single dilution of 1/1000 and incubated at 4°C overnight. 100 μL/well of rabbit anti-human IgG HRP Conjugate (1/5000) [Dako, Ely, UK] was added and incubated for 1 hour at room temperature. Plates were developed with o-phenyline-diamine [Sigma]-H_2_O_2 _and the reaction was stopped with 50 μL H_2_SO_4_. Plates were read at 490 nm. To generate an optical density (OD) cut-off value above which samples were deemed antibody positive, the distribution of OD values was fitted as the sum of two Gaussian distributions (assuming a narrow distribution of seronegatives and a broader distribution of seropositives) using maximum likelihood methods [37]. The mean OD of the Gaussian corresponding to the seronegative population plus three standard deviations was used as the cut-off for seropositivity. The normalised optical density in the different ELISA assays was considered as an indicator of the magnitude of antibody response in the analyses.

### Statistical analysis

Statistical analysis of data was performed using SPSS (version 14.0). Categorical variables were compared between groups by the Pearson Chi-square test or Fisher's Exact test. For non normally distributed numerical variables, the non-parametric Mann-Whitney rank sum test or Kruskal-Wallis H-test was used and the Interquartile range (IQR) was used to describe the spread around the median. For red blood cell polymorphisms, homozygotes and heterozygotes were separately compared with normal genotype as reference group. Multiple logistic and linear regression models were used to adjust estimates for potential confounders.

## Results

As previously reported for this population [4], parasite prevalence was similar in the dry and wet seasons (April and August, respectively), by microscopy (p = 0.49) and QT-NASBA (p = 0.10). Microscopic parasite prevalence and QT-NASBA parasite prevalence were not significantly related to age (table 1) [4]. Since all participants of the survey were derived from the general population and were apparently healthy, all parasite carriage can be considered to be asymptomatic. Our data indicate that 90.0% (135/150) of the asymptomatic parasite carriage occurs below the microscopic threshold for parasite detection. Only *P. falciparum *parasites were detected. Because there was no obvious seasonality or age dependency in parasite carriage, data from both cross sectional surveys were combined.

**Table 1:** The prevalence of *P. falciparum *infection by microscopy and QT-NASBA in relation to age

Parasite prevalence, % (n/N)	<5 years	5–15 years	>15 years	P-value
Microscopy, prevalence (n/N)	1.8 (11/608)	2.4 (18/744)	1.6 (22/1369)	0.42
Microscopy (selection)*	1.5 (2/137)	5.1 (8/156)	3.1 (5/160)	0.21
QT-NASBA	27.0 (37/137)	36.5 (57/156)	33.1 (53/160)	0.22

*Selection = only those individuals who also had valid QT-NASBA results. For eleven samples included in the QT-NASBA, no information on age was available.

In total, 100.0% (464/464) and 95.9% (445/464) of the samples were successfully genotyped for G6PD and α^+^-thalassaemia status, respectively. The frequency of G6PD deficiency was 9.7% (45/464; G6PD A heterozygotes) and 3.7% (17/464; G6PD A-homozygous/hemizygous). Of the G6PD A-individuals, 88.2% (15/17) were male, 11.8% (2/17) females. The frequency of α^+^-thalassaemia was 24.3% (108/445; αα/α- heterozygotes) and 3.6% (16/445; α-/α- homozygotes). Genotype polymorphisms were equally distributed among different age strata mentioned above (p = 0.80 and p = 0.71 for G6PD and α^+^-thalassaemia, respectively).

### Red blood cell polymorphisms and *P. falciparum *parasite carriage

Parasite prevalence detectable by microscopy was low and not related to red blood cell polymorphisms (table 2). The prevalence of parasitaemia by 18S QT-NASBA was much higher than that by microscopy, as previously described [4], but also showed no statistically significant association with either α^+^-thalassaemia or G6PD deficiency (table 2).

**Table 2:** The prevalence of *P. falciparum *infection and the frequency of G6PD and α^+^-thalassaemia variants

	*P. falciparum *parasite prevalence as detected by	
	Microscopy	QT-NASBA
**G6PD, % (n/N)**		
B	3.0 (11/366)	32.2 (118/366)
A	6.7 (3/45)	35.6 (16/45)
A-	0.0 (0/16)	37.5 (6/16)

α^+ ^– **thalassaemia, % (n/N)**		
αα/αα	3.7 (11/295)	30.5 (90/295)
αα/α-	2.0 (2/100)	40.0 (40/100)^¶^
α-/α-	0.0 (0/14)	14.3 (2/14)

G6PD B and α-thalassaemia αα/αα served as a reference group for the comparisons. ^¶^p = 0.08 when compared to α^+^-thalassaemia αα/αα. Only samples were included in this table that had results for both QT-NASBA and microscopy

### Sub-microscopic parasite carriage and *P. falciparum *specific immune responses

To compare the antibody response between sub-microscopic parasite carriers and parasite free individuals, we restricted our analyses to samples that had QT-NASBA results and excluded individuals who had microscopically detectable levels of asexual parasites (n = 14) and whose numbers did not allow separate analysis. As shown in table 3, the levels of antibodies to MSP-1 and MSP-2 were significantly higher for sub-microscopic parasitemic individuals compared to parasite-free individuals (p = 0.042 and p = 0.034, respectively), while the sero-prevalence of IgG antibodies to MSP-1 and MSP-2 was not significantly different between the groups (p = 0.086 and p = 0.33, respectively). For AMA-1 there was no statistically significant difference in prevalence or level of IgG antibody responses between sub-microscopic parasite carriers and parasite free individuals (p = 0.14). None of these associations were confounded by α^+^-thalassaemia or G6PD status.

**Table 3:** *P. falciparum *specific immune responses in parasite free individuals and individuals with sub-microscopic parasite densities by 18S QT-NASBA

	Parasite free individuals	Sub-microscopic parasite carriers	p-value
**MSP-1 IgG antibodies**			
prevalence, % (n/N)	68.0 (183/269)	76.7 (89/116)	0.086
median level (IQR)	0.26 (0.14 – 0.79)	0.33 (0.18 – 1.17)	0.042

**MSP-2 IgG antibodies**			
prevalence, % (n/N)	53.4 (134/251)	58.9 (66/112)	0.33
median level (IQR)	0.32 (0.13 – 0.59)	0.41 (0.19 – 0.72)	0.034

**AMA-1 IgG antibodies**			
prevalence, % (n/N)	61.0 (153/251)	65.2 (73/122)	0.44
median level (IQR)	0.34 (0.17 – 0.99)	0.48 (0.21 – 1.04)	0.14

Parasitaemia was detected by 18S QT-NASBA and microscopically confirmed parasite carriers were excluded from this table. The optical density in the ELISA was used as indicator of antibody level. P-values are obtained by χ^2^-test for antibody prevalence or Wilcoxon Rank-sum test for antibody level. The cut-off values for sero-positivity (antibody prevalence) for MSP-1, MSP-2 and AMA-1 were 0.171, 0.292 and 0.252, respectively.

Based on questionnaire data, we found no evidence of differences in recent symptomatic infections between parasite free and sub-microscopic parasite carriers. Between these groups, there was no statistically significant difference in self-reported illness (p = 0.74), antibiotic use (p = 0.73) or antimalarial use (p = 0.66) in the two weeks prior to sampling. In addition, body temperature at the moment of sampling was not different between sub-microscopic carriers and parasite-free individuals (p = 0.62).

## Discussion

In this cross sectional study, we investigated the possible relationship between sub-microscopic parasite carriage red blood cell polymorphisms, and malaria specific immune responses in an area of low malaria transmission intensity. Our main findings are that individuals carrying *P. falciparum *parasite densities below the microscopic detection limit (i.e. < 5 parasites/μL) had higher levels of IgG antibody response to asexual stage antigens MSP-1 and MSP-2 compared to parasite free individuals.

Parasite densities will fluctuate over time in chronic infections [38] and it is therefore possible that sub-microscopic parasite carriers had high density infections in the weeks or months prior to sampling. We nevertheless consider it unlikely that recent exposure to microscopically detectable levels of parasites can explain our findings. We did not find evidence for a higher occurrence of recent symptomatic infections in sub-microscopic parasite carriers and we consider asymptomatic high density infections unlikely in our study area of low endemicity where microscopically detectable infections in all age groups commonly result in symptomatic malaria and clinic attendance [29]. In addition, our study was conducted in a period when malaria transmission was extremely low as a consequence of prolonged drought [4], making recent (super-) infections unlikely. It has been previously reported that sub-microscopic parasite densities can remain at very low densities for several months [38] and therefore it is probable that the observed increased immune responses against blood stage parasites are the result of chronic sub-microscopic infections. The phenomenon that individuals with concurrent microscopic parasitaemia have higher antibody responses is well documented [39] but it is an important observation that this is also the case for sub-microscopic parasite densities. This suggests that low dose blood stage infections are capable of eliciting or maintaining humoral immune responses, as was previously shown for protective cell mediated immune responses [12]. Although our data are in line with a recently published hypothesis that low density infections contribute to sustained protection from malaria in various intervention studies conducted in areas of moderate to high transmission intensity [40], caution is needed when speculating about protective immunity. Asymptomatic chronic carriage of microscopically detected parasites has been associated with the induction of immune responses that may reduce the risk or rate of re-infection, parasite density, incidence of clinical malaria and severity of disease [10,11] but such a relation has never been studied with low density sub-microscopic infections. In our study area of low endemicity, it is likely that chronic infections are composed of one or a few clones and may therefore be poorly protective against infection with genetically distinct parasite strains [38]. Future longitudinal studies should confirm the immune boosting capacity of sub-microscopic parasite densities that we observe and determine if chronic infections at sub-microscopic densities are related to protective immunity.

This is the first report on the relationship between red blood cell polymorphisms and sub-microscopic parasitaemia. We hypothesized that the initial infection rates would be similar for different genotypes, while the time to reach densities that cause symptomatic malaria would be longer for G6PD deficient individuals [41] and individuals with α^+^-thalassaemia. Based on this hypothesis, we expected a higher prevalence of sub-microscopic parasite carriage in G6PD deficient and α^+^-thalassaemic individuals and a lower microscopic parasite carriage. Our data do not support this hypothesis. The number of individuals with microscopic levels of parasites was too small to allow any conclusions on a relation between asymptomatic microscopic parasite carriage and G6PD and α^+^-thalassaemia. Thus, sub-microscopic parasite carriage appears unrelated to G6PD deficiency or α^+^-thalassaemia.

## Conclusion

In summary, our data suggest a role for sub-microscopic parasite densities in eliciting or maintaining humoral immune responses without evidence for a modulating effect of G6PD deficiency or α^+^-thalassaemia. Longitudinal studies are required to determine the extent in which sub-microscopic parasites can generate (protective) immune responses. Ideally, these studies should include functional assays.

## Competing interests

The authors declare that they have no competing interests.

## Authors' contributions

TB, SS, RS, and CDconceived, designed and interpreted the results. SS, TB and CDconducted the study. AE, MA, SS, RK and SMperformed the experiments. TB, HM, SS and CDanalyzed the data; SS, TB, RS, CD, AM, MA and AEwrote the manuscript. All authors have read and approved the final manuscript.

## Pre-publication history

The pre-publication history for this paper can be accessed here:

http://www.biomedcentral.com/1471-2334/9/69/prepub
